# An outbreak of leptospirosis among kayakers in Brittany, North-West France, 2016

**DOI:** 10.2807/1560-7917.ES.2018.23.48.1700848

**Published:** 2018-11-29

**Authors:** Yvonnick Guillois, Pascale Bourhy, Florence Ayral, Mathilde Pivette, Anouk Decors, José Héctor Aranda Grau, Benoît Champenois, Célia Malhère, Benoît Combes, Céline Richomme, Marine Le Guyader, Lisa Antoinette King, Alexandra Septfons

**Affiliations:** 1 Santé publique France, Rennes, France; 2 Centre National de Référence de la leptospirose, Institut Pasteur, Paris, France; 3 VetAgro Sup, Université de Lyon, Marcy l'Etoile, France; 4 Office National de la Chasse et de la Faune sauvage, Auffargis, France; 5 Agence régionale de santé de Bretagne, Rennes, France; 6 Direction générale de l’alimentation, Ministère de l’agriculture et de l’alimentation, Paris, France; 7 Entente de lutte interdépartementale contre les zoonoses, Malzéville, France; 8 Anses (French Agency for Food, Environmental and Occupational Health and Safety), Nancy laboratory for rabies and wildlife, Malzéville, France; 9 Santé publique France, Saint-Maurice, France; 10 European Programme for Intervention Epidemiology Training, European Centre for Disease Prevention and Control, Stockholm, Sweden

**Keywords:** outbreak, leptospirosis, descriptive study, kayak, animal reservoir, France

## Abstract

In September 2016, a cluster of seven kayakers with clinical symptoms of leptospirosis with onset since July 2016 was reported to French health authorities. Human and animal investigations were undertaken to describe the outbreak, identify the likely place and source of infection and implement necessary control measures. We identified 103 patients with clinical symptoms of leptospirosis between 1 June and 31 October 2016 who lived in the Ille-et-Vilaine district in Brittany. Of these, 14 (including the original seven) reported contacts with the river Vilaine during the incubation period and were defined as outbreak cases: eight were confirmed by serology tests or PCR and six were probable without a laboratory confirmation for leptospirosis. All 14 cases were kayakers. Three distinct contamination sites were identified on a 30 km stretch of the river Vilaine. Nine cases reported having skin wounds while kayaking. None were vaccinated against leptospirosis. The outbreak was attributed to *Leptospira kirschneri* serogroup Grippotyphosa. Animal investigations did not allow identifying the possible reservoir. Leptospirosis outbreaks associated with freshwater sports are rare in temperate climates. The prevention of such outbreaks requires control of potential animal reservoirs in zones such as the Vilaine valley and that kayakers adopt the recommended individual prevention measures.

## Introduction

Leptospirosis is an important zoonotic disease caused by bacteria of the *Leptospira* genus after an incubation period of 4 to 21 days. The *Leptospira* genus contains 35 species, including 13 which are pathogenic and more than 300 serovars grouped into at least 24 serogroups [1,2].

Leptospirosis typically presents as a nonspecific acute febrile disease. In endemic areas, 60–70% of human infections have been reported as asymptomatic or pauci-symptomatic [3,4]. Symptomatic infections are generally non-severe (shivers, fever, headaches, muscular and joint pain). Severe clinical forms represent 5–10% of clinical forms and include kidney or liver failure, neurological complications and pulmonary and digestive bleeding. Death is reported for 5–15% of severe infections [5].

All wild and domestic mammals may be infected by *Leptospira* [6]. Among them, some species, especially rodents were reported as maintenance hosts of a given pathogenic *Leptospira*, carried in their kidney and excreted in urine for prolonged periods [7]. Rats (*Rattus* sp.) were reported as the primary carrier of the serogroup Icterohaemorrhagiae, responsible for most leptospirosis cases reported in humans [8]. Other wildlife species, e.g. coypu (*Myocastor coypus)*, muskrat (*Ondatra zibethicus)*, hedgehog (*Erinaceus europaeus)* and weasel (*Mustela nivalis*) have been described as renal carriers of pathogenic *Leptospira* in France or Western Europe and could act as a reservoir for human leptospirosis [9-11].

Bacterial transmission to humans occurs by direct contact with urine, blood or tissue of an infected animal, or most often by exposure to contaminated environment. *Leptospira* penetrate the human body through skin lesions or mucosa of the eyes, mouth, or nose.

In France, human leptospirosis is not a notifiable disease. According to the National Reference Centre for Leptospirosis (NRC), which is in charge of leptospirosis surveillance, mainland France has a high incidence (0.5 to one case per 100,000 inhabitants) compared with other industrialised countries with a similar temperate climate [12]. An increase in the number of confirmed cases in mainland France has been observed since 2014, with an incidence of one case per 100,000 inhabitants in 2014 and 2015, a figure twice as high as in 2011 [12,13]. Recent increases in the number of leptospirosis cases were also reported in several other European countries [14].

The epidemiology of leptospirosis is very different in French overseas territories where the climate is hot and humid, conditions that facilitate *Leptospira* survival. In these territories, incidence rates are 10 to 50 times higher than those seen in mainland France [12].

Leptospirosis outbreaks have previously been associated with recreational activities (canoeing, kayaking, water rafting, triathlon and swimming) that bring people into close contact with water contaminated with pathogenic leptospires [15]. Climatic conditions in intertropical zones promote outbreak situations [16,17]. In temperate zones such as mainland France, leptospirosis outbreaks are less frequent and generally limited in size [18].

On 9 September 2016, a kayak club in a rural community in Brittany, North-West France, alerted health authorities about a cluster of kayakers who had developed leptospirosis symptoms since July 2016. Starting from 16 September, seven patients had been identified, including three with a laboratory confirmation of leptospirosis. Symptom onset in the patients was between 4 July and 15 September 2016.

The identification of seven kayakers from the same club with leptospirosis symptoms suggested an outbreak linked to kayaking on the river Vilaine, the main river in Brittany.

Human and animal investigations were undertaken to describe the outbreak, identify the likely place and source of infection and implement necessary control measures.

## Methods

### Investigation of human leptospirosis cases

A descriptive study was undertaken and completed by microbiological analyses. Cases were identified in two steps. First, patients with leptospirosis-like symptoms were actively sought. In a second step, part of them were classified as outbreak cases using laboratory and epidemiological criteria.

#### Identification of patients with leptospirosis-like symptoms

**Figure 1:**
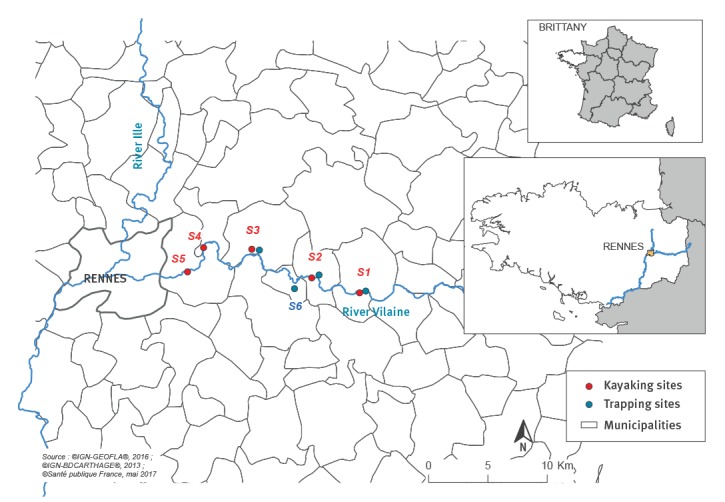
Kayaking sites, leptospirosis outbreak, Brittany, France, 2016

Retrospective and prospective identification was undertaken of patients living in the Ille-et-Vilaine administrative district of Brittany who had developed symptoms compatible with leptospirosis between 1 June and 31 October 2016. Multiple data sources were used:

- six kayak clubs on a 30 km suspected stretch of the river Vilaine upstream of Rennes, capital city of the Brittany region (Figure 1),
- general practitioners in the valley surrounding the Vilaine,
- the clinical laboratories of the five closest hospitals,
- two large private laboratories that carry out leptospirosis analyses for blood samples taken at private and hospital laboratories.

S1, S2, S3, S4, S5 are kayaking sites on the suspected stretch of the river Vilaine. S1, S2, S3, S4 are kayaking sites where animals were trapped for wildlife investigations. S6 is a site of the suspected stretch of the river Vilaine where animals were trapped. It is separate and distinct from the kayaking sites.

For the first two sources, patient identification was directly based on reported leptospirosis-like symptoms: shivers, fever, headache, muscle and joint pain. For the last two sources, a person who had been sampled for laboratory confirmation of leptospirosis between 1 June and 31 October 2016 was assumed to be a patient with symptoms compatible with leptospirosis, whatever the results of the laboratory analyses and the analytical method used. The same patient could be identified via multiple sources. Duplicates were identified and deleted before data analyses.

#### Case definition

The previously identified patients were too many for us to investigate all of them. A part of them was contacted by telephone to assess their exposure to the river Vilaine. With the rising workload, we concentrated mainly on patients for whom information was more readily available; these were the patients identified through kayak clubs, local GPs and laboratories of local hospital. These contacted patients were either classified as cases or excluded using laboratory and epidemiological criteria. Outbreak cases were defined in the following manner:

- Confirmed case: a patient exposed to the suspected stretch of the river Vilaine during the incubation period (within three weeks before symptom onset) with a laboratory confirmation for leptospirosis (PCR, ELISA or microscopic agglutination test (MAT));
- Probable case: a patient exposed to the suspected stretch of the river Vilaine during the incubation period without a laboratory confirmation for leptospirosis;
- Excluded case: patient not exposed to the suspected stretch of the river Vilaine during the incubation period.

The non-classified patients were patients who had not been investigated by telephone regarding their exposure to the water of the river Vilaine. They were all patients (regardless of laboratory confirmation) with leptospirosis-like symptoms identified by the two large private laboratories and the university teaching hospital.

#### Descriptive study

Confirmed and probable cases were questioned by telephone to collect demographic characteristics, clinical and biological information, recreational and professional exposures during the incubation period (within three weeks before symptom onset) and information on individual protective measures used against leptospirosis.

The geographical distribution of cases’ home in relation to kayak clubs was described with geocoding (BDAdresse).

#### Microbiological analyses

Only patients who reported contacts with the river Vilaine were asked for a blood sample for microbiological analyses by the NRC. Serum samples were systematically subjected to serology tests at the NRC: an in-house IgM ELISA and the MAT [19]. MAT was performed using 24 *Leptospira* antigens to identify the serogroup of the infecting strain with a threshold of ≥1:100 [12].

For patients with symptoms for less than a week, total genomic DNA from plasma and urine were tested for the presence of pathogenic *Leptospira* by qPCR [19]. *Leptospira* genospecies were characterised by partial 16S rRNA gene sequencing [20].

#### Additional analyses for non-classified patients

Additional analyses were performed to assess the potential presence of leptospirosis cases linked to the outbreak among the non-classified patients. We selected the non-classified patients who were, like the cases, younger than 60 years. They were supposed to be of an age compatible with close contact with water when kayaking on the river Vilaine (e.g. bathing and kayak rolls). Among them, we sought patients living in the same areas as cases. Their living areas were assessed through their home postcodes and the addresses of their general practitioner and medical laboratory. Their distance to the kayak clubs of the river Vilaine was also described.

### Environmental investigations

Agricultural activities, livestock and wildlife along the suspected stretch of the river were described qualitatively. We also described the river Vilaine’s hydrology and the weather conditions of the summer 2016 (source: Météo-France).

### Wildlife investigations

We conducted investigations to identify *Leptospira* circulating in wild animals living in the area where human cases had been exposed. During 14 consecutive days following the outbreak (October 2016), semi aquatic rodents (coypu and muskrat) were captured alive with large (100 × 35 × 35 cm) single-catch traps and were euthanised in the context of pest control. Small mammals (wood mice (*Apodemus sylvaticus*) and bank voles (*Myodes glareolus*)) were also trapped during two 3-day periods in October and November 2016 using medium (50 × 15 × 15 cm) or small (28 × 9 × 9 cm) catch traps. All trapped animals were euthanised in accordance with the French Animal Protection Law and Directive 2010/63/EU of the European Parliament and of the Council on the protection of animals used for scientific purposes (identification code of the approval (29/09/2016) project AP AFIS#2939–20160106142231; name of the ethics committee: Cometh).

The field researchers were trained to sample the dead animals following predetermined guidelines. Kidney tissues were removed immediately after euthanasia and frozen pending further analyses at the Laboratoire des Leptospires (Marcy L'Etoile, France). Molecular analysis including PCR, rrs gene typing and variable-number tandem repeats (VNTR) were performed according to previously published laboratory procedures [9].

Serological status of animals was determined using MAT with a panel of antigens representing 14 ubiquitous and locally prevalent serogroups. A seroreactivity ≥ 1:100 directed against at least one serogroup was associated with a past or recent exposure to *Leptospira* of the analysed animal. We used the highest titre directed against one unique serogroup to define the predominant serogroup [21].

## Results

### Human investigations

#### Case description

In total, we identified 103 patients residing in the Ille-et-Vilaine administrative district, with symptoms compatible with leptospirosis between 1 June and 31 October 2016. Seven of them were initially identified kayakers. The 96 remaining patients were identified from the hospital clinical laboratories and the two large private laboratories.

**Figure 2:**
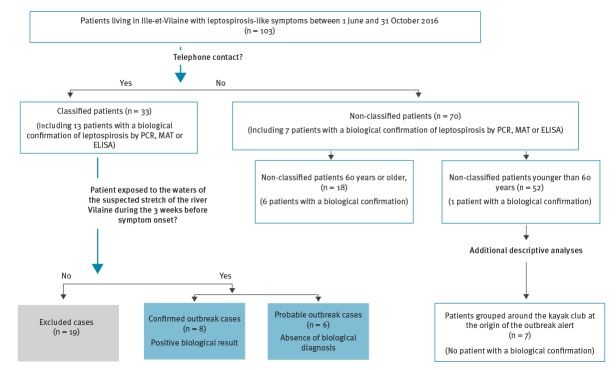
Classification of patients with symptoms compatible with leptospirosis, Brittany, France, 1 June–31 October 2016 (n = 103)

Thirty-three (32%) of the patients with leptospirosis-like symptoms were interviewed by telephone and excluded or classified as confirmed or probable cases (Figure 2). Nineteen of them were excluded because they had not had contact with water of the river Vilaine. Fourteen patients, including the seven original kayakers, were outbreak cases: eight confirmed cases and six probable cases (Figure 2).

MAT: microscopic agglutination test.

**Figure 3:**
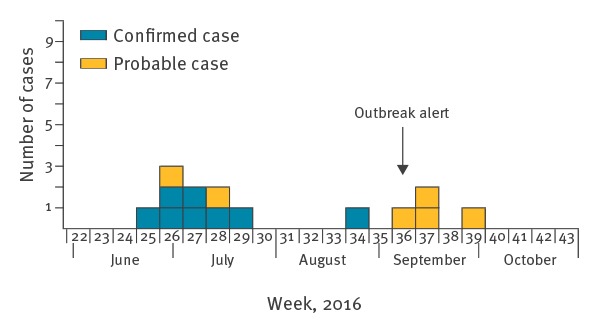
Cases of leptospirosis by week of onset, Brittany, France, 1 June–31 October 2016 (n = 14)

In terms of geographical distribution, all cases lived grouped in the Vilaine valley upstream of Rennes. Eleven cases lived less than 5 km from a kayak club and eight lived less than 5 km from the club that gave rise out the outbreak alert. This latter is located in site S3 (Figure 1). Most cases were male (86%). The median age was 18 years (range: 11–60). The 14 cases reported symptom onset between 22 June and 28 September 2016 (Figure 3).

All cases reported generalised symptoms (fever, shivering, tiredness, headaches, muscle or joint pain). Nine cases reported digestive symptoms. Each of the additional following symptoms (neurological, respiratory, ocular, renal and skin/mucous membranes) was reported by one to three cases. Three cases, of whom two were confirmed, were hospitalised. Two were hospitalised for 2 days, the third for 9 days. Among the remaining 11, at least four (two confirmed) consulted the accident and emergency services of local hospitals. Nine cases reported the presence of skin wounds, deep or superficial. No information was collected about the protection of wounds, their cleansing and disinfection after contact with the Vilaine water. No case was vaccinated against leptospirosis.

All cases were kayakers from four different clubs of the Vilaine valley upstream of Rennes. In the three weeks before symptom onset, four cases had kayaked on the suspected stretch of the river every day, three cases several times a week, the seven others three times or less. All cases reported close contact with the river water via bathing or undertaking kayak rolls. Half of them had practiced kayak-polo which also leads to close contact with the river water. Eight cases had kayaked at a single site on the suspected river stretch: S1, S3 or S5 (Figure 1). Two cases reported kayaking in a swimsuit and T-shirt. Five reported wearing a short wetsuit and seven a full-body wetsuit. None wore gloves. No other known risk exposure was reported. Only two cases had travelled outside of mainland France during their incubation period but none in a leptospirosis endemic area.

### Microbiological analyses

Analyses by the NRC allowed typing of the bacteria for five of the eight confirmed cases. MAT identified a single serogroup for four cases: Grippotyphosa. For only the fifth case, the genospecies was identified by sequencing: *L. kirschneri.* The corresponding serogroup could not be determined. However, the *L. kirschneri* species is common to several serogroups including serogroup Grippotyphosa [22]. The outbreak can thus reasonably be attributed to the Grippotyphosa serogroup.

The reservoir of the outbreak strain could have been present at several sites or along the entire suspected stretch of the river because three of the five cases frequented only one single nautical activity site each: S1, S3 or S5 (Figure 1).

### Additional analyses concerning non-classified patients

Seventy patients could not be classified as a case, including seven patients with a positive biological test. Fifty-two of these non-classified patients were younger than 60 years like the cases. We considered that people of that age could be assumed to practice kayaking with close contact with the river water (kayak rolls, kayak polo…). One of these 52 patients had laboratory-confirmed leptospirosis. Twenty-three patients younger than 60 years had a residential postcode in common with one or more of the cases. Among them, we identified only seven patients living close to the kayak club at the origin of the outbreak alert: two lived in that same rural municipality, five had consulted a general practitioner in the municipality and six went to a local laboratory less than 2 km from that municipality in order to be tested for leptospirosis.

Based on this age and geographical distribution, we assume that the proportion of additional cases among the non-classified patients was low.

### Wildlife investigations

Thirty-eight wild animals were trapped in four sites along the suspected stretch of river (26 coypus, two muskrats, six wood mice and four bank voles), including 27 captured close to the kayak club at the origin of the alert: S1 (Figure 1).

For nine animals captured in sites S1, S2 and S3 (Figure 1), detection of *Leptospira* DNA by PCR indicated ongoing infections. Three pathogenic and intermediate *Leptospira* species were identified [23]. *Leptospira borgpetersenii was identified in* one muskrat, *L. wolffii* in two coypus and *L. interrogans* in two coypus, two wood mice, one muskrat and one bank vole (Table). Molecular typing identified *L. interrogans* related to the serogroup Djasiman Gurungi from a muskrat sample.

**Table:** Captured wild animals (species) exposed to or infected with *Leptospira,* Brittany, France, 2016 (n = 38)

Species	Total captured	Exposed animals^a^	Infected animals^b^
		Positive/tested	Positive/tested
Wood mice *(Apodemus sylvaticus)*	6	2/5	2/6
Bank vole *(Myodes glareolus)*	4	0/2	1/4
Coypu *(Myocastor coypus)*	26	20/26	4/26
Muskrat *(Ondatra zibethicus)*	2	2/2	2/2
**Total**	**38**	**24/35**	**9/38**

^a^ Idenitified by the microscopic agglutination test. Serogroups tested: Icterohaemorrhagiae, Australis, Autumnalis, Ballum, Bataviae, Canicola, Cynopteri, Grippotyphosa, Hebdomadis, Panama, Pomona, Pyrogenes, Sejroe and Tarassovi.

^b^ Idenitifed by PCR.

A positive seroreactivity observed by MAT suggested exposure to *Leptospira* in 24 of the 35 tested animals (Table). The serological profiles suggested mainly exposure to the serogroups Australis (n = 18) and Sejroe (n = 4). Based on the laboratory results, exposure to the serogroup Grippotyphosa was suggested for a single animal, a wood mouse captured at the site S2.

### Environmental investigations

Different livestock and agricultural activities were present along the suspected stretch of the river Vilaine. Milk-producing cattle herds were the principal identified livestock.

A large increase in the numbers of semi-aquatic wild rodents (coypu, muskrat) has been reported around the nautical activity site at the origin of the outbreak alert since the cessation of trapping activities by volunteers certified as trappers at the end of 2015. The municipality of the kayak club at the origin of the alert did not manage to identify new volunteers to pursue trapping activities before the outbreak.

Upstream of Rennes, the river Vilaine is characterised by low water levels and low water flow during summers. Moreover, the slate subsoil of the valley is impermeable. Consequently, the suspected river stretch may be very sensitive to the run-off of animal waste (including urines potentially contaminated by *Leptospira*) during rain showers.

The summer of 2016 was the fourth hottest summer since 1996. The mean temperature was 18.9 °C compared with 18.1 °C for summers of the period from 1996 to 2015.

### Immediate control measures

The kayak club at the origin of the alert was supposed to host a kayak-polo tournament on 17 and 18 September 2016 with 300 to 400 participants from all over France. Local governmental authorities cancelled the tournament and all water sports were banned from 16 September in the nautical base opposite the club: S3 (Figure 1).

Kayak clubs situated on the suspected stretch of the river were informed of the outbreak, about the disease (reservoir, causative agent and clinical symptoms), individual protective measures and the need for a rapid medical consultation in the presence of symptoms. The information was transmitted via leaflets and a signpost erected on the site where water sports were banned.

With the help of the local governmental authorities, the municipality of the closed nautical activity site identified new certified trappers who were asked to trap and quickly kill coypus and muskrats.

On 21 October, the ban of the nautical base was lifted in view of resumed trapping of semi-aquatic wild rodents and decreased water temperature of the river which offered less favourable conditions for the survival of *Leptospira*.

## Discussion and conclusions

This investigation identified in mainland France an outbreak of 14 leptospirosis cases among kayakers on the river Vilaine (Brittany). Outbreaks of similar sizes and associated with recreational water sports have been reported in intertropical zones but appear to be rare in temperate zones such as mainland France [18,24-26].

The microbiological analyses suggest that the outbreak cases were caused by the serogroup Grippotyphosa. The cases reported several known potential risk factors. Only half of the identified cases used a full-body wetsuit for kayaking although wearing long suits may be a strong protective factor against infection by *Leptospira* [4]. Nine of the 14 cases reported kayaking with skin wounds, which allow *Leptospira* to enter into the body. No case was vaccinated despite French recommendations that adults who regularly practise freshwater sports such as swimming, kayaking and canoeing should be vaccinated. Nevertheless, it is to be noted that the vaccine is effective only against the Icterohaemorrhagiae serogroup which causes approximately one third of leptospirosis cases. Therefore, vaccination would have not prevented this outbreak [27].

The animal reservoir remained unidentified. Despite investigation of various animal species and a maximised detection sensitivity using live capture and immediate sampling after death, *Leptospira* species and MAT profiles obtained from captured rodents and small mammals were not consistent with the ones observed in humans. The MAT profile suggesting Grippotyphosa exposure in a single wood mouse was uncertain because the MAT correctly predicts the infecting serogroup in only 46–86% of cases [21,28]. In Germany in 2007, the investigation of an outbreak of *L. kirschneri* serogroup Grippotyphosa identified common voles as the reservoir for humans [29]. Muskrats could also be the reservoir of serogroup Grippotyphosa for humans according to previous work suggesting Grippotyphosa exposure among muskrats in North West France (GEDUVER project on sustainable management of vertebrate predators in rural environment; data not shown) and in Belgium in 2012 [30].

Insufficient sample size and diversity of captured wild animals could explain why we could not identify the potential animal reservoir of *L. kirschneri* for this outbreak. The trapping activities that were put in place rapidly after the alert and before the wildlife investigations might have reduced the number of animals that could be trapped and analysed. Here, only two of the 38 animals sampled were muskrats, which is most probably not a sufficient reflection of the diversity of *Leptospira* that could be carried in this animal species. Moreover, the animal investigations focused on wildlife because kayakers and local authorities reported a large and recent increase in the semi-aquatic rodent population. Therefore, we did not test the livestock present along the river, which can also be a reservoir of the bacteria.

Nevertheless, the diversity of *Leptospira* species observed in this survey is consistent with what has previously been reported in wildlife [9]. It remains possible that a limited circulation of the outbreak strain among wild fauna at the suspected stretch of the river could have been sufficient to cause the outbreak if the strain was particularly virulent [6,31].

At least three distinct sites of contamination of cases were identified along the suspected stretch of the river Vilaine. Immediate control measures were put in place only around the nautical base of the initially implicated kayak club. However, no other cases were identified at any of the sites after the initial control measures were set up. This investigation also underlined the difficulty to identify objective criteria for when to end control measures put in place on a river in the context of a leptospirosis outbreak. For example, screening large bodies of freshwater for *Leptospira* is not believed to be useful for guiding public health authorities in decisions regarding the safe recreational use of water because failure to detect pathogenic *Leptospira* provides no valid information about water safety. In addition, the public health risk associated with a positive result cannot be assessed because infection depends on the dose and type of exposure [16,25].

The summer 2016 in the Ille-et-Vilaine administrative district was warmer than usual in this area. Moreover, the river Vilaine is associated with low water levels and low water flow during summer. An increase in the semi-aquatic rodent population was also reported by kayakers using the implicated stretch of river before the outbreak. The outbreak thus occurred in an environmental context that was likely to facilitate contamination of the river and bacterial survival in the water [32]. In the context of global climate change, such environmental conditions could be reproduced more regularly in temperate zones like mainland France and result in more regular leptospirosis outbreaks [23].

In terms of study limitations, the clinical description of the outbreak cases was based solely on patient-reported symptoms. While information about generalised symptoms seemed robust, information about renal, neurological and skin/mucous membrane symptoms seemed less certain. The severity of illness could thus be underestimated for certain cases. However, the low hospitalisation rate and emergency room visits suggest that most patients had mild symptoms. Our investigation did not identify individual risk factors associated with this outbreak. A case–control study including serological testing of controls would have been necessary to get this information. The true number of leptospirosis cases associated with the outbreak was probably underestimated. Despite active case finding using four data sources, not all identified patients were interviewed and classified. The used data sources would not have identified mild or asymptomatic cases without a medical consultation. It is also possible that kayakers could have hesitated to notify their symptoms for fear of seeing their kayaking activities suspended in connection with the closure of a stretch of the river Vilaine.

Following this investigation, several recommendations can be made to prevent the occurrence of another such outbreak. Kayakers, even in temperate zones, need to be well informed about the risk of leptospirosis, the nature of the disease, the importance and benefits of a rapid medical consultation and the benefits and limits of protective measures: individual protection equipment, vaccination and wound hygiene measures (cleansing and disinfection). It is also important to raise awareness among all healthcare workers about the diagnosis, especially when risk factors for leptospirosis such as water sport activities are identified.

In the absence of an identified animal reservoir for this outbreak, studies could be continued among wild and domestic animals along the stretch of the Vilaine, in particular muskrats and the livestock previously described as susceptible to the Grippotyphosa serogroup [30,33]. This outbreak occurred in the context of an increase in the semi-aquatic rodent population following cessation of trapping activities. It serves as a reminder that rodent species known for *Leptospira* carriage need to be controlled close to freshwater sporting sites [11,30,34,35].

The private and clinical laboratories were sufficient to identify outbreak cases in this investigation. A surveillance system based on these two data sources could thus be put in place in zones where leptospirosis outbreaks occur such as the valley of the river Vilaine.
